# Distribution of *Pseudocercospora* species causing Sigatoka leaf diseases of banana in Uganda and Tanzania

**DOI:** 10.1111/ppa.13105

**Published:** 2019-10-11

**Authors:** J. N. Kimunye, E. Were, F. Mussa, A. Tazuba, K. Jomanga, A. Viljoen, R. Swennen, F. K. Muthoni, G. Mahuku

**Affiliations:** a International Institute of Tropical Agriculture, PO Box 7878, Kampala, Uganda; b Department of Plant Pathology, Stellenbosch University, Private Bag X1, Matieland 7602, South Africa; c International Institute of Tropical Agriculture (IITA), Dar es Salaam, PO Box 34441, Tanzania; d International Institute of Tropical Agriculture (IITA), c/o Nelson Mandela African Institution of Science and Technology, Nelson Mandela Road, Arusha, Tanzania; e Laboratory of Tropical Crop Improvement, KU Leuven, Willem De Croylaan 42, 3001 Leuven, Belgium; f IITA-Arusha, Arusha, PO Box 10 Duluti, Tanzania

**Keywords:** black Sigatoka, disease severity, *Musa* spp, niche expansion, *Pseudocercospora fijiensis*

## Abstract

Sigatoka leaf diseases are a major constraint to banana production. A survey was conducted in Tanzania and Uganda to assess the distribution of *Pseudocercospora* species and severity of Sigatoka leaf diseases. *Pseudocercospora* species were identified using species-specific primers. Sigatoka-like leaf diseases were observed in all farms and on all cultivars, but disease severity varied significantly (*P* < 0.001) between countries, districts/regions within countries, altitudinal ranges and banana cultivars. In all regions except Kilimanjaro, *P. fijiensis*, the causal agent of black Sigatoka, was the only pathogen associated with Sigatoka disease. *Mycosphaerella musae* was associated with Sigatoka-like symptoms in Kilimanjaro region. Black Sigatoka disease was more severe in Uganda, with a mean disease severity index (DSI) of 37.5%, than in Tanzania (DSI = 19.9%). In Uganda, black Sigatoka disease was equally severe in Luwero district (mean DSI = 40.4%) and Mbarara district (mean DSI = 37.9%). In Tanzania, black Sigatoka was most severe in Kagera region (mean DSI = 29.2%) and least in Mbeya region (mean DSI = 11.5%). *Pseudocercospora fijiensis*, the most devastating sigatoka pathogen, was detected at altitudes of up to 1877 m a.s.l. This range expansion of *P. fijiensis*, previously confined to altitudes lower than 1350 m a.s.l. in East Africa, is of concern, especially for smallholder banana farmers growing the susceptible East African Highland bananas (EAHB). Among the banana varieties sampled, the EAHB, FHIA hybrids and Mchare were the most susceptible. Here, the loss of resistance in Yangambi KM5, a banana variety previously resistant to *P. fijiensis*, is reported for the first time.

## Introduction

Banana and plantain (*Musa* spp.) are an important staple, providing food and income to millions of people in Africa. Most bananas produced and consumed in the African Great Lakes region are the East Africa Highland bananas (EAHB), a genetically related collection of plants that belong to the Mutika/Lujugira subgroup of triploid AAA bananas (Kitavi *et al*., 2016). Over 85% of bananas in East Africa are produced by smallholder farmers (FAO, 2016). Despite its importance as a staple, production of the crop is declining (FAO, 2016). This, in part, is due to diseases such as Sigatoka leaf diseases caused by fungal pathogens in the genus *Pseudocercospora* (Jones, 2000; Marĺn *et al*., 2003; Churchill, 2011).

Three *Pseudocercospora* species are associated with Sigatoka leaf diseases: *P. fijiensis* (previously *Mycosphaerella fijiensis*) that causes black Sigatoka, *P. musae* (previously *M. musicola*) causing yellow Sigatoka, and *P. eumusae* (previously *M. eumusae*) that causes eumusae leaf spot. The epidemiology of the three diseases is similar. Infection of young banana leaves is initiated by asexual spores that are dispersed over short distances by rain splash, or ascospores (sexual spores) carried by wind for several kilometres (Rieux *et al*., 2014). Dispersal of the fungus over longer distances is by humans through the movement of infected planting materials and leaves that are used as packaging materials. When conditions (temperature, humidity and rainfall) are favourable for disease development, susceptible plants can be completely defoliated in a relatively short period (Jones, 2000; Marĺn *et al*., 2003). This results in small and poorly filled bunches and irregular ripening, with yield reduction ranging from 35% to 80% (Mobambo *et al*., 1993).

Yellow Sigatoka caused by *P. musae* was the first of the Sigatoka disease complex to be reported in Africa, first in Uganda in 1938, then Zanzibar in 1939, and later in other African countries (Stover, 1962). In Uganda, the disease was more prevalent at higher altitudes (above 1350 m above sea level, a.s.l.) and cooler temperatures (below 13.8 °C). Disease incidence was higher on introduced ABB varieties than EAHB, thus the disease was considered of low importance (Johanson *et al*., 2000). *Pseudocercospora eumusae* causing eumusae leaf spot was reported from southern and southeast Asia in the late 1990s (Carlier *et al*., 2000). In Africa, *P. eumusae* is present in Nigeria (West Africa) where it is reported to co-occur with *P. fijiensis* (Zandjanakou-Tachin *et al*., 2009). *Pseudocercospora eumusae* has not been reported from East Africa.

Black Sigatoka was first reported in Fiji in 1963, and it rapidly spread to become one of the most important pathogens of banana globally (Jones, 2000). In Africa, black Sigatoka was first reported in Gabon, West Africa in 1978 (Frossard, 1980). The first report in East Africa was in 1987 from the islands of Pemba, Tanzania (Dabek & Waller, 1990), after which it spread to other neighbouring countries in the Great Lakes region of Africa (Tushemereirwe & Waller, 1993). Cultural strategies, such as the removal of infected leaves, are available to smallholder farmers for managing Sigatoka diseases (Jones, 2000). In commercial plantations, Sigatoka diseases are managed using an intensive spray regime of fungicides that often accounts for over 30% of production costs (Churchill, 2011).

In Africa, black Sigatoka is most prevalent at mid-altitude areas below 1350 m a.s.l., and was previously not found above this altitude (Tushemereirwe *et al*., 1993; Mouliom-Pefoura *et al*., 1996; Johanson *et al*., 2000). However, the adaptation range of *P. fijiensis* has gradually expanded to higher and cooler altitudes (Mouliom-Pefoura *et al*., 1996; Erima *et al*., 2017). The changing climate (i.e. increase in temperatures and less rainfall; Omambia *et al*., 2012) is affecting banana production, pathogen survival and spread (distribution) as well as disease severity (Mouliom-Pefoura *et al*., 1996; Erima *et al*., 2017). It is therefore postulated that the distribution and severity of *P. fijiensis* in Uganda and Tanzania may have changed. Studies of Sigatoka leaf diseases in the African Great Lakes region were last conducted in Uganda in 2012 (Erima *et al*., 2017). The study documented the presence or absence of Sigatoka (Tushemereirwe *et al*., 1993; Johanson *et al*., 2000; Erima *et al*., 2017), without recording disease severity. There are no recent studies to document incidence and severity of Sigatoka in Tanzania. It is important to document the incidence and severity of Sigatoka diseases and use this information to identify disease hotspots, the most prevalent pathogen and to guide deployment of management strategies.

This study was conducted to: (i) determine the incidence and severity of Sigatoka leaf diseases in Tanzania and Uganda; and (ii) identify *Pseudocercospora* species associated with Sigatoka leaf diseases in the two countries. This information will be used to guide local banana breeding programmes on where to screen hybrids and varieties for resistance to Sigatoka leaf diseases and variety deployment.

## Materials and methods

### The study area

**Figure 1:**
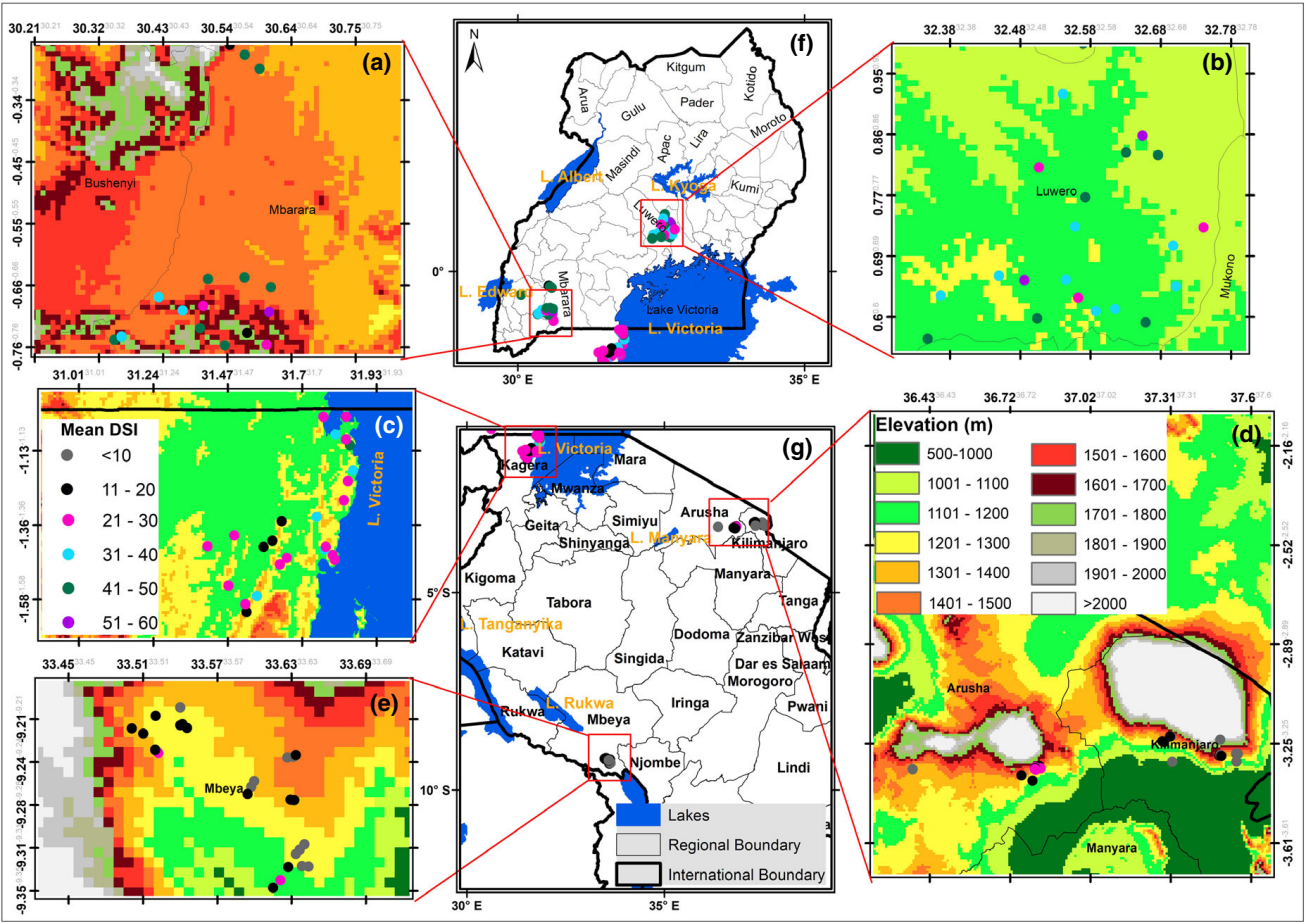
Distribution and severity of Sigatoka in two regions in Uganda and three regions in Tanzania. A and B are Mbarara and Luwero districts, respectively, in Uganda, while C, D and E are Kagera, Kilimanjaro and Mbeya regions, respectively, in Tanzania. Mean disease severity index (%) is denoted by colour-coded circles embedded on the map.

Surveys of Sigatoka leaf diseases were conducted in five geographic locations in East Africa (Table 1; Fig. 1). In Tanzania the administrative areas under study are referred to as regions while in Uganda they are known as districts. Historical weather data on daily minimum and maximum temperatures from 1980 to 2016 in Uganda, and from 1980 to 2010 in Tanzania, was obtained from the department of meteorology in each country. Where weather station data was not available it was extracted from Climate Hazards Group Infrared Precipitation with Stations (CHIRPS) satellite data (Funk *et al*., 2015).

**Table 1:** Study sites, location, samples collected and incidence of *Pseudocercospora fijiensis* in Uganda and Tanzania.

Country	District/region	Latitude	Longitude	Altitude (m a.s.l.)^a^	No. of farms	No. of samples	Samples positive for *P. fijiensis*^b^
Uganda	Luwero	32.6277	0.8711	1077–1243	23	132	102 (77%)
	Mbarara	30.6545	–0.6072	1411–1877	20	143	100 (70%)
Tanzania	Kagera	31.8050	–1.3296	1148–1394	24	140	132 (94%)
	Kilimanjaro	36.6830	–3.3869	1210–1530	25	159	0 (0%)
	Mbeya	33.4608	–8.9094	1064–1455	32	295	236 (80%)

a Altitude classification: low, 1000–1200 m a.s.l.; mid, 1201–1500 m a.s.l.; high, >1500 m a.s.l.

b The identity of the fungus was established using PCR with species-specific primers.

### Survey methodology and disease rating

Surveys were conducted from April to July 2016. Forty-three banana farms were surveyed in Uganda, and 81 farms in Tanzania. The selected farms were at least 5 km apart. Geographical coordinates for each farm were recorded using a hand-held eTrex 10 global positioning system (GPS) device (Garmin). Farmers were interviewed to obtain information on source of planting materials, banana variety and production system and management practices. On each farm, 15 plants at the preflowering stage were randomly selected and evaluated for Sigatoka leaf disease. The number of standing leaves was counted, and each leaf was visually rated for the stage of symptom development (Fouré, 1987), and disease severity assessed on a 0–6 scale (Gauhl, 1994). The disease severity index (DSI) was computed as:

$$DSI=\frac{\sum nb}{(N-1)T}\times 100.$$

where *n* = number of leaves in each grade, b = grade (0–6), *N* = number of grades used in the scale (7), *T* = total number of leaves scored.

Leaves with typical Sigatoka-like leaf disease symptoms at stages 3–6 (Fouré, 1987) were collected from each plant that was evaluated, placed between two sheets of newspaper, labelled and transported to the laboratory for species identification.

### DNA extraction

Fungal DNA was extracted directly from banana leaves with Sigatoka-like disease lesions at stages 3–6 using the protocol described by Mahuku (2004), with some modifications as follows. Three or four lesions were excised from the leaf using a sterile scalpel, and the cutting mat cleaned with 70% ethanol between samples. The lesions were then ground in a sterile mortar using a pestle in 700 μL of prewarmed (65 °C) TES extraction buffer (0.2 M Tris-HCl, pH 8; 10 mM EDTA, pH 8; 0.5 M NaCl; 1% w/v SDS; 1% w/v sodium sulphite) containing proteinase K (50 μgmL^−1^). The rest of the extraction was performed as per the protocol except that precipitation in isopropanol was done overnight. The DNA pellet was then resuspended in 100 μL TE buffer, and the DNA integrity determined using a NanoDrop 2000C spectrophotometer (Thermo Fisher Scientific Inc.). DNA concentrations for each sample were adjusted to 50 ng μL^−1^ for PCR analysis.

### Identification of *Pseudocercospora* species

*Pseudocercospora* species were identified using species-specific primers developed by Johanson & Jeger (1993) and Arzanlou *et al*. (2007). PCR conditions were as described by Johanson & Jeger (1993). The primers used were MF137/R635 for *P. fijiensis*, MMactF2/MMactRb for *P. musae*, and ActF/MEactR for *P. eumusae*. Amplifications were performed in a TC-512 thermocycler (Techne) and consisted of an initial denaturation step at 95 °C for 4 min; followed by 35 cycles of 1 min at 94 °C, 1 min at 68 °C and 1 min at 72 °C; with a final 3 min extension step at 72 °C. The annealing temperature for primers MMactF2/MMactRb and ActF/MEactR was 60 °C. The amplification products were kept at 4 °C until further analysis. Controls included DNA from *Pseudocercospora* species obtained from the CBS culture collection in Utrecht, Netherlands (CBS numbers: *P. fijiensis* 120258/CIRAD 86, *P. musae* 116634/X42 and *P. eumusae* 121383/CIRAD744). The PCR products were resolved on a 1.5% w/v agarose gel in 1 × TAE buffer at 150 V for 45 min. Gel images were captured using the GBOX gel documentation system (SYNGENE).

To identify the cause of Sigatoka-like symptoms in Kilimanjaro, the *actin* gene of 20 samples from Kilimanjaro, three from Mbeya and three *P. fijiensis* samples from Morogoro (reference samples) regions was amplified using general actin primers ACTF/ACTR (Arzanlou *et al*., 2007). PCR products were sequenced using the same primers, the sequences aligned using GENEIOUS PRIME software, and used in BLAST searches against the NCBI sequence database.

### Data analysis

Molecular data was scored as presence (1) and absence (0) of the expected fragment size. The percentage of positive samples was computed from the total number of samples per region. Pearson’s chi-square test was used to determine the existence of significant association between cultivar distribution and altitudes. Analysis of variance (ANOVA) was computed using GENSTAT v. 15 software (VSN International Ltd) to determine statistically significant association between Sigatoka disease severity and banana cultivar (genome group) at different altitude ranges (<1200, 1201–1400, 1401–1600 and >1601 m a.s.l.). Means were separated using Fisher’s least significant difference (LSD) test at *P* < 0.05. Maps to show the distribution and severity of Sigatoka were generated using ARCGIS software (ESRI). Meteorological data from each region was used to analyse effect of climatic trends on Sigatoka leaf disease incidence and severity. The Mann–Kendall trend nonparametric (MK) test was used to detect temperature (minimum and maximum) trends. The Sen’s slope estimator was used to determine the magnitude of observed (decreasing or increasing) trends. XLSTAT software was employed to analyse the trends with *P*-values derived at 95% confidence levels.

## Results

### Farming and banana production systems

Most banana farms (63%) were situated in mid-altitude areas at 1200–1500 m a.s.l., while 26% of the farms were in high altitudes (>1501 m a.s.l.). The rest (11%) of the farms were in low altitude areas (<1200 m a.s.l.). Bananas were produced on farms ranging from 0.2 to 4 ha, mainly for home consumption, and excess sold in local markets. All plantations were established using suckers from the owner’s old farm or from neighbours. None of the farmers used inorganic fertilizers; instead, they relied on organic fertilizers, mostly livestock and chicken manure. None of the farmers interviewed used fungicides to control Sigatoka. Most of the farmers (85%) relied on good sanitation practices (removal of lower infected and senescent leaves and excess suckers, and weeding) for managing Sigatoka. The leaves and extra suckers were used as animal feed.

In all farms visited, banana was grown as mixtures of EAHB, plantain, dessert, roasting and brewing bananas. EAHBs were dominant in Uganda, whereas in Tanzania Mchare and Kimalindi varieties were most prevalent in Kilimanjaro region. In Mbeya region, plantains were more prevalent followed by EAHB and Kimalindi. EAHB and FHIA hybrids, FHIA 17 and FHIA 23, were the most common banana varieties in Kagera region. The distribution of banana types was significantly (*P* < 0.001) associated with altitude (Table 2). More banana types were observed at lower altitudes of Uganda, but the EAHB was more predominant across altitudes (Table 2). In both countries under study, banana was intercropped with coffee, cassava, beans, groundnuts, taro and maize.

**Table 2:** Pearson’s chi-square test of association between prevalence of banana types and altitude in Uganda and Tanzania.

Country	Altitude (m a.s.l.)	Frequency of different banana type/variety							
		EAHB	Plantain	Sukari Ndizi	ABB	FHIA	Dessert	Yangambi KM5	Mchare
Uganda	<1200	281	6	11	12	6	11	3	N/A^b^
	1201–1400	25	0	0	3	0	3	0	N/A
	1401–1600	136	9	23	0	0	19	2	N/A
	>1600	76	4	6	0	0	7	0	N/A
Tanzania	<1200	78	5	5	3	33	12	14	1
	1201–1400	152	78	21	9	67	82	34	25
1401–1600	19	14	6	2	1	25	0	40
>1600^a^	—	—	—	—	—	—	—	—

Association between altitude and banana type/variety: Uganda, *P* < 0.001, χ^2^ = 57.3, critical value 42.3, d.f. = 18; and Tanzania, *P* < 0.001, χ^2^ = 57.3, critical value 190.4, d.f. = 14.

a No sites above 1601 m a.s.l. were visited in Tanzania.

b Cultivar was not encountered in the survey area.

### Incidence and severity of Sigatoka leaf diseases

**Figure 2:**
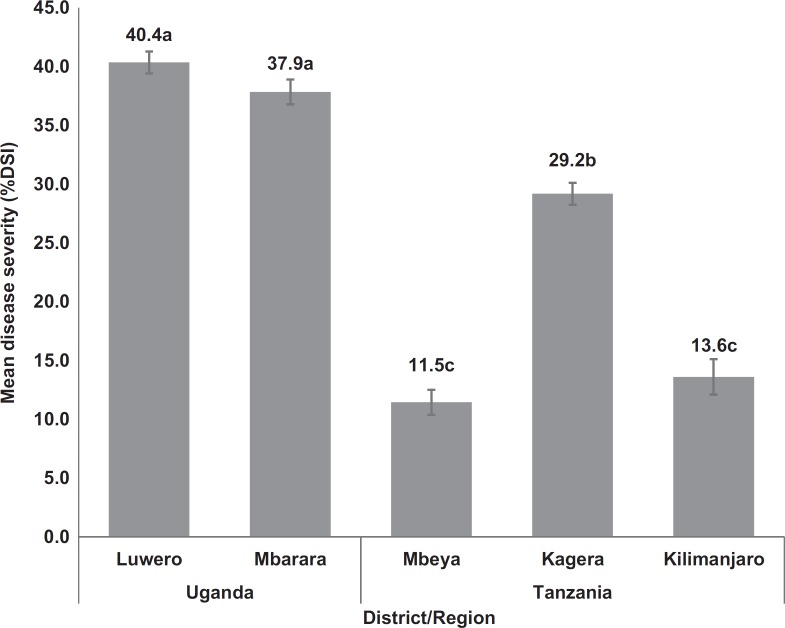
Mean Sigatoka severity index in different regions surveyed in Uganda and Tanzania. Numbers followed by the same letter are not statistically different.

**Figure 3:**
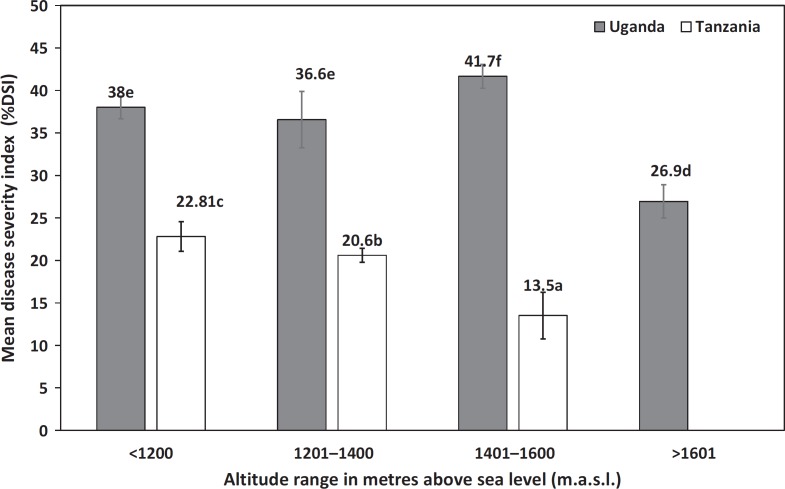
Mean Sigatoka severity index across the different altitude ranges in Uganda (Luwero and Mbarara districts) and Tanzania (Kilimanjaro, Mbeya and Kagera regions). Numbers followed by the same letter are not statistically different. Sites with areas <1200 m a.s.l. are Luwero district, Kagera and Mbeya regions; 1201–1400 m a.s.l., Luwero district, Mbeya, Kilimanjaro and Kagera regions; 1401–1600 m a.s.l., Mbeya and Kilimanjaro regions and Mbarara district; 1601–1800 m a.s.l., Mbarara district; and >1800 m a.s.l., Mbarara district.

Sigatoka-like symptoms were observed in all sites surveyed (Fig. 1a,b,c,d,e). Disease symptoms progressed to the necrotic stage at all altitudes in all study areas except in Kilimanjaro. Disease severity was significantly (*P* < 0.001) higher in Uganda with a mean DSI of 39.3%, while in Tanzania mean DSI was 20.2%. No significant differences in mean DSI were observed between Luwero and Mbarara districts (Fig. 2) despite their difference in altitude. Similarly, mean DSI did not vary significantly between Kilimanjaro and Mbeya regions. In general, disease severity decreased with increasing altitude. However, this was not the case for Mbarara district, where disease was more severe at altitudes of 1401–1600 m a.s.l. (Fig. 3). The symptoms in Kilimanjaro region were different from the typical Sigatoka symptoms and were characterized by chlorotic and necrotic lesions with smoky patches on the upper leaf surface. The dry leaf areas lacked the characteristic grey centres associated with late stages of black and yellow Sigatoka (Fig. S1a). The lower leaf surface had irregular black blotches (Fig. S1b) consistent with banana leaf speckle caused by *Mycosphaerella musae*.

### Identification of *Pseudocercospora* species

Amplification of DNA from pure *P. fijiensis* using the species-specific primer pair MF137/R635 gave the expected 1000 bp fragment (Fig. S2). These primers were then used to amplify DNA extracted directly from banana leaves with Sigatoka symptoms. The presence of *P. fijiensis* was confirmed in all regions and altitudes except Kilimanjaro (Table 1). The samples from Kilimanjaro also did not amplify with primers specific to *P. musae* or *P. eumusae*. The reference cultures obtained from CBS produced expected amplicons for *P. musae* and *P. eumusae*, confirming the specificity of these primers for the target pathogens (Fig. S3a,b). Amplification of a selected set of isolates from Kilimanjaro, Mbeya and Morogoro regions using the *actin* gene primers gave the expected 820 bp fragment. A nucleotide BLAST of sequences obtained from the Kilimanjaro region samples revealed that 65% of the samples had 90–99% homology to *M. musae* strain CBS121386, accession LC121215.1, confirming the visual diagnosis of leaf speckle. The rest of the samples shared identities with other fungi such as *Phoma medicaginis, Phoma nigrificans* and *Amycosphaerella africana* (Table S1). Known *P. fijiensis* isolates collected from Mbeya and Morogoro regions were included as reference and these confirmed PCR identification of *P. fijiensis* (Table S1).

### Cultivar response to black Sigatoka

Banana cultivars are given different names by farmers depending on the region where they are grown. For simplicity, the cultivars were grouped based on genome complement as AAA (EAHB-cooking/brewing), ABB (Pisang Awak and Bluggoe), AAB (Sukari Ndizi), AA (Mchare), AAAB (FHIA hybrids, FHIA 17 and FHIA 23), AAA (dessert (Kimalindi, Bogoya), plantain and Yangambi KM5). All cultivars inspected had Sigatoka symptoms but disease severity was significantly different (*P* < 0.001) between cultivars or genome groups. The EAHB and FHIA hybrids were the most susceptible type of bananas in both Uganda and Tanzania. Disease severity between genome groups was significantly (*P* = 0.007) associated with altitude range (Table 3). In Tanzania, disease severity for all genome groups decreased with increase in altitude except in FHIA hybrids, where disease severity was highest at 1201–1400 m a.s.l. Such a trend was not observed in Uganda where some banana types had high disease severities at 1401–1600 m a.s.l. However, disease severity declined at altitudes >1600 m a.s.l. in all banana types (Table 3). Yangambi KM5 was considered highly resistant to *P. fijiensis*, but in this study symptoms on this cultivar progressed to necrotic stages, like those on the susceptible EAHB and Mchare cultivars.

**Table 3:** Mean Sigatoka severity index (%) for different banana types at different altitude ranges in Uganda and Tanzania.

Country	Altitude (m a.s.l.)	EAHB	Plantain	Sukari Ndizi	ABB	FHIA hybrids	Dessert types	Yangambi KM5	Mchare
Uganda	<1200	41.67 ± 1.05	34.06 ± 7.21	46.27 ± 5.3	32.98 ± 5.1	27.38 ± 7.21	32.63 ± 5.32	18.89 ± 10.19	—^a^
	1201–1400	36.82 ± 3.53	—	—	51.77 ± 10.19	—	32.34 ± 10.19	—	—
	1401–1600	42.87 ± 1.51	44.01 ± 5.88	40.57 ± 3.68	—	—	43.27 ± 4.05	14.29 ± 12.48	—
	>1600	31.16 ± 2.02	15.94 ± 8.82	19.14 ± 7.21	—	—	16.85 ± 6.67	—	—
Tanzania	<1200	23.67 ± 1.99	10.6 ± 7.89	15.36 ± 7.89	31.34 ± 10.19	24.13 ± 3.07	21.16 ± 5.09	22.75 ± 4.71	35.9 ± 17.65
	1201–1400	22.16 ± 1.24	14.32 ± 1.97	7.13 ± 3.85	3.36 ± 5.88	39.71 ± 2.16	12.68 ± 1.95	—	22.94 ± 3.53
	1401–1600	15.13 ± 4.05	9.27 ± 4.71	8.74 ± 7.21	9.91 ± 12.48	—	10.76 ± 3.53	20.67 ± 3.03	14.3 ± 2.79
	>1600^b^	—	—	—	—	—	—	—	—
LSD		3.18							
Cultivar type × Altitude		0.007							

a Banana type not sampled at that altitude.

b No bananas were sampled above 1600 m a.s.l. in Tanzania.

### Analysis of climatic trends across sites

**Figure 4:**
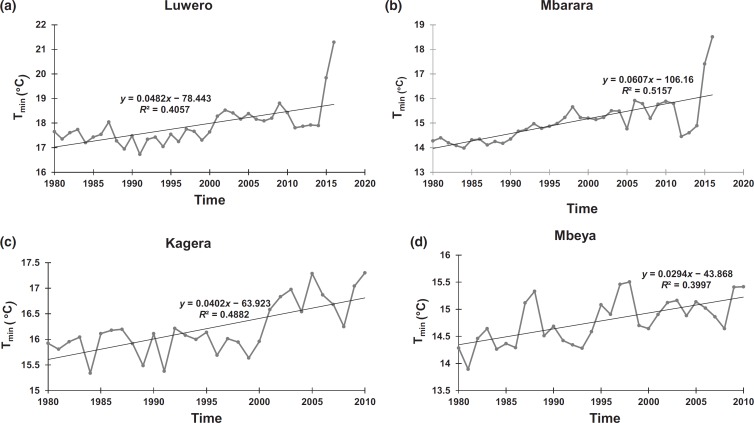
Annual average minimum temperature trends in two districts, (a) Luwero and (b) Mbarara in Uganda (1980–2016) and two regions, (c) Kagera and (d) Mbeya in Tanzania (1980–2010). In all countries, the general trend is an increase in minimum temperature (T_min_) with time.

**Figure 5:**
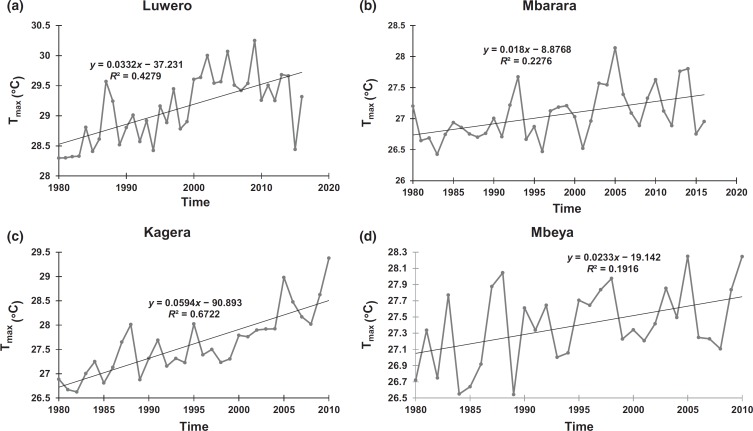
Annual average maximum temperature trends in two districts, (a) Luwero and (b) Mbarara in Uganda (1980–2016) and two regions, (c) Kagera and (d) Mbeya in Tanzania (1980–2010). The trends reveal that the regions under study are getting warmer (increasing T_max_).

The annual mean temperature (T_min_ and T_max_) in 1980–2016 for Uganda, and 1980–2010 for Tanzania were 14.8–17.9 °C and 27.6–29.1 °C, respectively. Luwero district was the warmest while Mbarara district had the lowest T_max_ (Table 4). The lowest mean T_min_ was recorded in Mbeya region (14.8 ± 0.4 °C) while Luwero district had the highest mean (Table 4). Mann–Kendall statistic revealed a significant (*P* < 0.05) increase in T_min_ and T_max_ in all sites over the same period. Mbarara district experienced the highest rate of T_min_ increase at 0.06 °C per year while Mbeya region had the lowest rate at 0.03 °C per year (Fig. 4). Similarly, T_max_ significantly increased in all study areas. The highest rate of T_max_ increase was observed in Kagera region at 0.06 °C per year and the least increase of 0.02 °C per year in Mbarara district and Mbeya region (Fig. 5).

**Table 4:** Mean temperature and rainfall values with standard deviation of annual aggregates of meteorological variables analysed in four sites for the period 1980–2016 (Uganda) and 1980–2010 (Tanzania).

Country	District/region	Altitude (m a.s.l.)	Annual T_min_ (°C)			Annual T_max_ (°C)		
			Minimum	Maximum	Mean	Minimum	Maximum	Mean
Uganda	Luwero	1077–1243	16.7 ± 0.8	21.3 ± 0.8	17.9 ± 0.8	28.3 ± 0.6	30.3 ± 0.6	29.1 ± 0.6
	Mbarara	1411–1877	13.9 ± 0.9	18.5 ± 0.9	15.1 ± 0.9	26.4 ± 0.4	28.1 ± 0.4	27.1 ± 0.4
Tanzania	Mbeya	1064–1455	13.9 ± 0.4	15.5 ± 0.4	14.8 ± 0.4	26.5 ± 0.5	28.3 ± 0.5	27.4 ± 0.5
	Kagera	1148–1394	15.3 ± 0.5	17.3 ± 0.5	16.2 ± 0.5	26.6 ± 0.7	29.4 ± 0.7	27.6 ± 0.7

## Discussion

Black Sigatoka, caused by *P. fijiensis*, was first reported in Tanzania in 1987 and in Uganda in 1989 (Dabek & Waller, 1990; Tushemereirwe & Waller, 1993). At that time the disease was present only at altitudes below 1350 m a.s.l. and at temperatures above 15 °C (Johanson *et al*., 2000). *Pseudocercospora musae*, the cause of yellow Sigatoka, was most prevalent at altitudes above 1350 m a.s.l. and temperatures below 14 °C (Tushemereirwe *et al*., 1993, 2004; Johanson *et al*., 2000). In this study, *P. fijiensis* was the only *Pseudocercospora* species associated with Sigatoka leaf diseases and was detected at elevations as high as 1800 m a.s.l. This finding confirms the hypothesis that *P. fijiensis* distribution has changed. Occurrence of the pathogen in all surveyed areas suggests the presence of an environment conducive to *P. fijiensis* infection and development, the presence of susceptible cultivars and an adequate source of inoculum. Previous reports of *P. fijiensis* presence at high altitudes have been made in Uganda (Erima *et al*., 2017), Colombia (Belalcázar, 1991), where the pathogen was detected at 1600 m a.s.l. 10 years post-incursion, and in Costa Rica, where *P. fijiensis* was observed to have expanded from 900 to 1500 m a.s.l. within five years (Marĺn *et al*., 2003). These observations reveal a gradual adaptation of the pathogen to cooler conditions associated with higher altitudes. The expanding adaptation range of *P. fijiensis* into higher elevations more suited for EAHB threatens production of this important staple crop in the region. Continuous surveillance to monitor pathogen spread is needed to better document factors leading to range expansion.

The change in temperature over the past 20 years might have contributed to the displacement of *P. musae* in Uganda and Tanzania. Erima *et al*. (2017) reported that minimum temperatures in the high-altitude areas in Uganda (>1400 m a.s.l.) have increased by more than 1 °C over the last two decades. Dispersal and proliferation of *P. fijiensis* is favoured by warm conditions, 25–27 °C, and high humidity, 90–100% (Churchill, 2011), thus giving it a competitive advantage over *P. musae*, which thrives well under cooler conditions (18.4 °C) (Mouliom-Pefoura *et al*., 1996; Rocha *et al*., 2012).

High black Sigatoka severities were observed at altitudes of 1401–1600 m a.s.l. for most of the banana types surveyed in Uganda. This observation suggests either an environment that is more conducive to *P. fijiensis* proliferation or emergence of more virulent patho-types better adapted to higher altitudes (Legler *et al*., 2012). Recombination arising from sexual mating facilitated by the occurrence of both mating types has been reported for *P. fijiensis* (Conde-Ferráez *et al*., 2010). Studies to elucidate the structure and diversity of *P. fijiensis* populations from these regions should be conducted to shed more light on possible reasons for habitat expansion and increased disease severity.

Changes in temperature and rainfall patterns may alter a pathogen’s growth, development rate, survival, dispersal and virulence or aggressiveness (Jones, 2000; Churchill, 2011; Legler *et al*., 2012). Analysis of historical weather data from the study sites revealed that temperatures have increased over the past three decades, creating an environment more conducive for *P. fijiensis* proliferation. A gradual increase in minimum temperature was observed in Mbarara district from 14.2 °C in 1980 to 18.5 °C in 2016, creating more suitable conditions for *P. fijiensis* to infect bananas. The 15 °C minimum temperature threshold for *P. fijiensis* proliferation was attained in 1997; thus, it is possible that the pathogen may have gradually established in the high-altitude areas with increasing minimum temperatures, leading to the currently observed dominance. The absence of black Sigatoka at high elevations in Mbarara during the earlier studies was attributed to either temperature-based altitudinal threshold for *P. fijiensis* proliferation or absence of the pathogen in this part of Uganda (Tushemereirwe *et al*., 1993; Johanson *et al*., 2000).

The emergence of environments suitable to *P. fijiensis* calls for the deployment of Sigatoka management strategies that involve resistant germplasm and good agronomic practices such as soil fertility regimes, proper drainage, mat management and general field sanitation. Fungicides are not an option for resource-limited smallholder banana farmers in Africa. They rely on a range of cultural practices to reduce inoculum load and minimize pathogen proliferation. Such practices include weeding, pruning of old diseased leaves and removal of excess suckers, which are often fed to animals (Sivirihauma *et al*., 2017). When not fed to livestock, infected leaves are left on the ground, where they can be a source of inoculum for up to 20 weeks. This period can be reduced to 3–6 weeks by cutting leaves into small pieces and heaping or composting (Gauhl, 1994; Villalta & Guzmán, 2005). Cutting and piling up leaves hastens decomposition and reduces the release and dispersal of spores (Gauhl, 1994; Jones, 2000). In this study, it was observed that farmers left cut leaves on the ground. There is a need to create awareness and teach small-holder farmers effective methods to manage pruned infected banana leaves to minimize subsequent infection by *P. fijiensis*.

Sigatoka-like symptoms observed in the Kilimanjaro region were identified as being caused by *M. musae*, resulting in mycosphaerella speckle. This pathogen has been observed and reported on banana (Jones, 2000) but is considered economically not important. The level of severity observed on banana in this region warrants further studies to establish its impact on banana production. Although *P. fijiensis* was not detected in Kilimanjaro region, the pathogen is present in other areas close to this region. Kilimanjaro region has experienced significant decline in precipitation over the last 37 years (Muthoni *et al*., 2018), which could have rendered the area less conducive for *P. fijiensis* proliferation. Furthermore, soils in Kilimanjaro region are volcanic and known to be rich in silicon (Zech *et al*., 2014), which is reported to modulate development and establishment of *P. fijiensis* (Kablan *et al*., 2011). The high levels of silicon in the soils in this region might also be contributing to the absence of *P. fijiensis*. Further studies are required to establish whether *P. fijiensis* is indeed absent from this region.

All banana cultivars grown in the Great Lakes region were susceptible to black Sigatoka (Tushemereirwe *et al*., 1993, 2004; Johanson *et al*., 2000; Erima *et al*., 2016). Previously, the cultivar Yangambi KM5 was resistant, exhibiting a hypersensitive response to *P. fijiensis* infection (Carlier *et al*., 2003). In this study, Sigatoka lesions progressing to stage 6 of infection were observed on Yangambi KM5 growing in Kagera region, and no differences in disease severity were observed between Yangambi KM5 and the black Sigatoka-susceptible EAHB and Mchare varieties. This might suggest the presence of *P. fijiensis* pathotypes capable of overcoming resistance in Yangambi KM5. Isolates belonging to mating type 1 and 2 of *P. fijiensis* have been observed under field conditions and occurring in equal ratios in Mexico and Brazil (Conde Ferráez *et al*., 2010; Queiroz *et al*., 2013). Similar observations were made in this study (data not presented), pointing to the possibility of frequent sexual reproduction in nature. It is therefore possible that new *P. fijiensis* pathotypes capable of overcoming resistance in Yangambi KM5 arose through sexual recombination. Although breakdown of Sigatoka resistance in Yangambi KM5 has been reported in Costa Rica, Puerto Rico and Cameroon (Mouliom-Pefoura, 1999; Irish *et al*., 2013; Escobar-Tovar *et al*., 2015), to the best of the authors’ knowledge, this is the first report of breakdown of resistance in this variety in East Africa. Population genetic studies and virulence profiles are required to further characterize isolates from this region. This will provide a better understanding of evolution of new pathotypes and its significance for managing black Sigatoka disease.

This study confirms that *P. fijiensis*, the cause of black Sigatoka of banana, is widespread in Uganda and Tanzania. Previously confined to lower altitudes, *P. fijiensis* has moved and adapted to higher elevations previously considered unsuitable. Analysis of historical weather data confirmed that temperatures have increased. The yellow Sigatoka pathogen was not detected, even at higher altitudes where the pathogen was previously present, signifying a change in pathogen profiles. This study also showed that *M. musae* was the pathogen associated with leaf symptoms in Kilimanjaro region. Studies are needed to document the significance of *M. musae* on banana production in this region. An integrated disease management strategy that includes use of resistant varieties and good agronomic practices should be deployed to safeguard the livelihood of smallholder farmers in the Great Lakes region of Africa.

## Supporting Information

**Figure S1**. Typical banana leaf spot symptoms observed in Kilimanjaro region. (a) Upper leaf surface with smoky patches. The necrotic lesions lack grey centres. (b) Black irregular patches on the lower leaf surface with moisture.

**Figure S2**. Gel showing amplification of DNA from infected banana leaf samples using the primer pair MF137/R635 giving the expected amplicon of 1000 bp. Lane 1, *Pseudocercospora fijiensis* DNA (CBS 120258); lane 2, blank (ddH_2_O); lanes 3–8, samples from Mbeya and Kagera regions; lanes 9–11, samples from Kilimanjaro region; lanes 12–20, samples from Mbarara and Luwero districts; lanes 21 and 22, CBS isolates of *P. musae* and *P. eumusae*, respectively; lane 23, blank (ddH_2_O); L, DNA ladder (2-log DNA ladder (New England Biolabs)).

**Figure S3**. Gel showing amplification of DNA from infected leaf samples using primer pairs MMactF2/MMactRb and ActF/MEactR giving the expected amplicon of 200 and 630 bp, respectively. (a) Amplification with *Pseudocercospora musae*-specific primers MMactF2/MMactRb. Lane 1, *P. musae* DNA (CBS 116634); lane 2, blank (ddH_2_O); lanes 3–8, samples from Mbeya and Kagera regions; lanes 9–11, samples from Kilimanjaro region; lanes 12–20, samples from Mbarara and Luwero districts; lanes 21 and 22, CBS isolates of *P. fijiensis* (CBS 120258) and *P. eumusae* (CBS 121383), respectively; lane 23, blank (ddH_2_O); L, DNA ladder (2-log DNA ladder (New England Biolabs)). (b) Amplification with *P. eumusae*-specific primers ActF/MEactR. Lane 1, *P. eumusae* DNA (CBS 121383); lane 2, blank (ddH_2_O); lanes 3–8, samples from Mbeya and Kagera regions; lanes 9–11, samples from Kilimanjaro region; lanes 12–20, samples from Mbarara and Luwero districts; lanes 21 and 22, CBS isolates of *P. fijiensis* and *P. musae*, respectively; lane 23, blank (ddH_2_O); L, DNA ladder (2-log DNA ladder (New England Biolabs)).

**Table S1**. Summary of NCBI BLAST search results with sequences for the *actin* gene for samples from Kilimanjaro, Morogoro and Mbeya regions in Tanzania.
