# Supplementary figures and images for: Distribution of Pseudocercospora species causing Sigatoka leaf diseases of banana in Uganda and Tanzania
Source: Plant Pathol. 2019 Oct 11;69(1):50–9. doi: 10.1111/ppa.13105 (PMC6919302; doi:10.1111/ppa.13105)

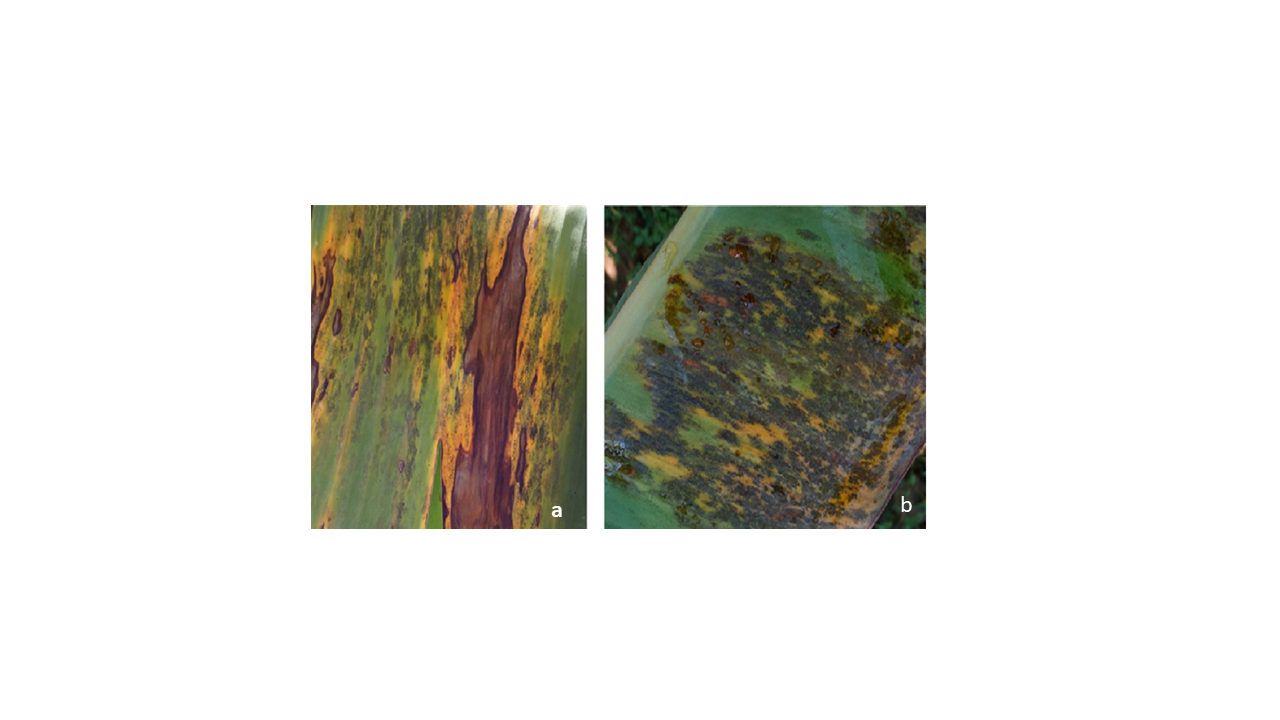

Supplement: Supplementary file 1 [file PPA-2019-PPA-13105-s1.tif]

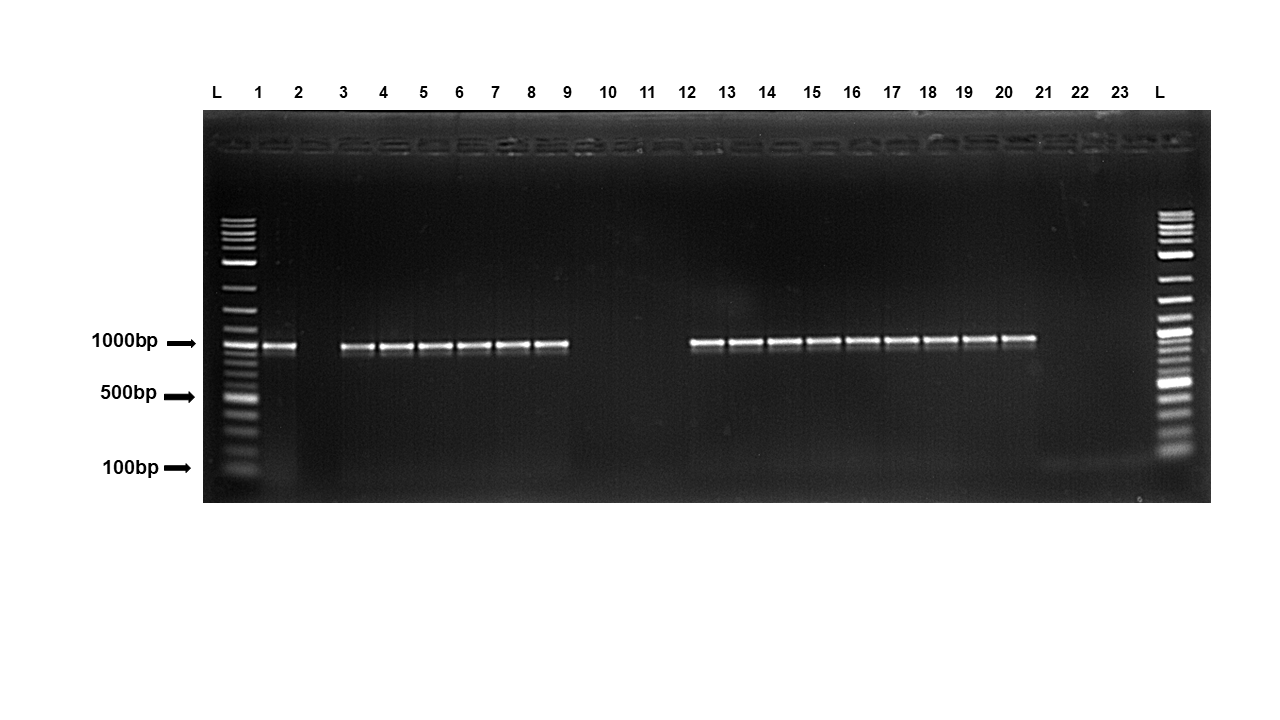

Supplement: Supplementary file 2 [file PPA-2019-PPA-13105-s2.tif]

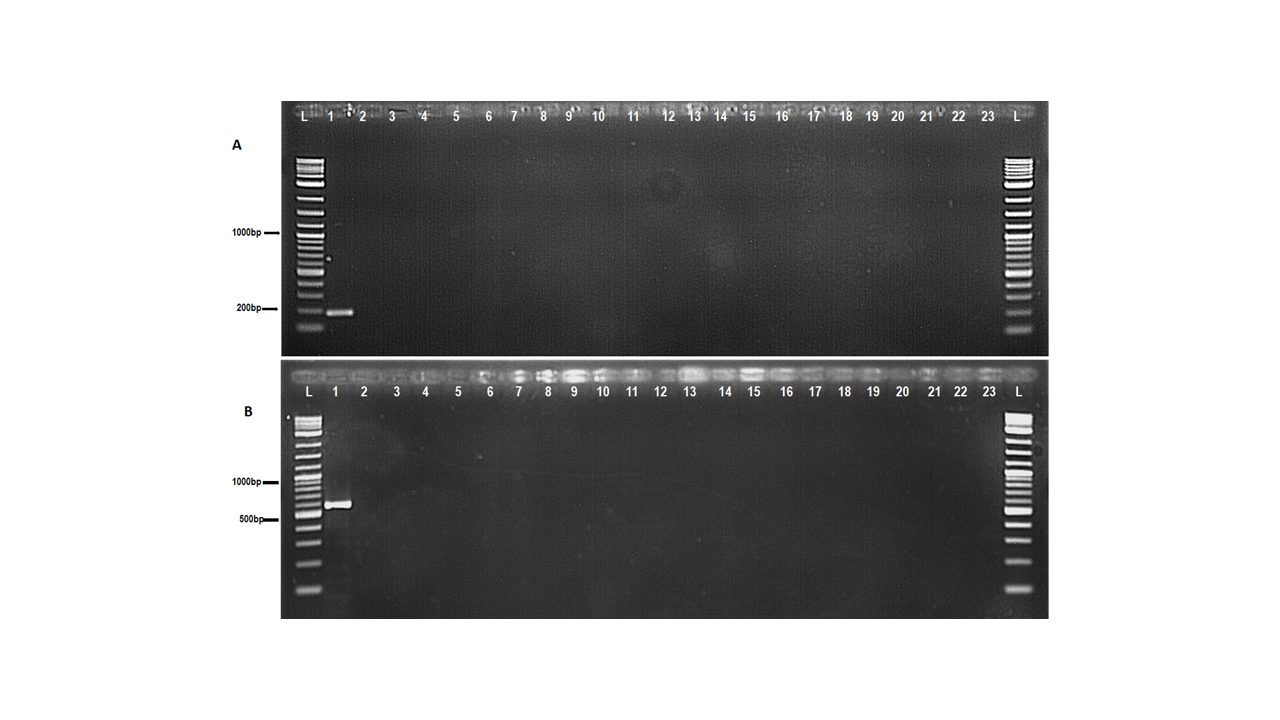

Supplement: Supplementary file 3 [file PPA-2019-PPA-13105-s3.tif]
