# Supplementary material for: Distribution of Pseudocercospora species causing Sigatoka leaf diseases of banana in Uganda and Tanzania
Source: Plant Pathol. 2019 Oct 11;69(1):50–9. doi: 10.1111/ppa.13105 (PMC6919302; doi:10.1111/ppa.13105)
Supplement: Supplementary file 4 [file PPA-2019-PPA-13105-s4.docx]

| Serial no. | Sample ID | Sample origin | Sequence based Identification | Gene bank accession number | E value | % Identity | Query coverage % |
| --- | --- | --- | --- | --- | --- | --- | --- |
| 1 | TE21 | Kilimanjaro | *Mycosphaerella musae Actin gene* | LC121215.1 | 2.92E-145 | 99 | 99.3 |
| 2 | TE17 | Kilimanjaro | *Mycosphaerella musae Actin gene* | LC121215.1 | 0 | 99.5 | 99.3 |
| 3 | TE7 | Kilimanjaro | *Mycosphaerella musae Actin gene* | LC121215.1 | 0 | 97.5 | 100 |
| 4 | AR69 | Kilimanjaro | *Mycosphaerella musae Actin gene* | LC121215.1 | 0 | 92.2 | 99.1 |
| 5 | AR21 | Kilimanjaro | *Phoma medicaginis* | HM157445.1 | 0 | 93.4 | 98 |
| 6 | AR34 | Kilimanjaro | *Phoma medicaginis* | HM157445.1 | 0 | 93 | 72 |
| 7 | AR48 | Kilimanjaro | *Phoma nigrificans* | AY748980.1 | 0 | 91.4 | 84 |
| 8 | AR185 | Kilimanjaro | *Mycosphaerella musae Actin gene* | LC121215.1 | 0 | 99.5 | 98.8 |
| 9 | AR173 | Kilimanjaro | *Mycosphaerella musae Actin gene* | LC121215.1 | 2.90E-143 | 86.9 | 84.1 |
| 10 | AR170 | Kilimanjaro | *Mycosphaerella musae Actin gene* | LC121215.1 | 0 | 90.3 | 97.8 |
| 11 | AR88 | Kilimanjaro | *Mycosphaerella musae Actin gene* | LC121215.1 | 0 | 93.8 | 98.8 |
| 12 | AR58 | Kilimanjaro | *Mycosphaerella musae Actin gene* | LC121215.1 | 4.68E-166 | 92.8 | 89.7 |
| 13 | AR45 | Kilimanjaro | *Mycosphaerella musae Actin gene* | LC121215.1 | 0 | 98.2 | 98.4 |
| 14 | AR36 | Kilimanjaro | *Mycosphaerella musae Actin gene* | LC121215.1 | 0 | 94.4 | 66.2 |
| 15 | AR22 | Kilimanjaro | *Mycosphaerella musae Actin gene* | LC121215.1 | 0 | 90.1 | 93 |
| 16 | AR29 | Kilimanjaro | *Amycosphaerella africana* | LC121211.1 | 7.00E-107 | 95.6 | 95 |
| 17 | AR78 | Kilimanjaro | *Amycosphaerella africana* | LC121211.1 | 5.00E-169 | 92.4 | 91 |
| 18 | AR14 | Kilimanjaro | *Mycosphaerella musae Actin gene* | LC121215.1 | 5.00E-169 | 92.8 | 89 |
| 19 | AR56 | Kilimanjaro | *Phoma nigrificans* | AY748980.1 | 0 | 93.3 | 73 |
| 20 | AR66 | Kilimanjaro | *Phoma nigrificans* | AY748980.1 | 0 | 91.9 | 97 |
| 21 | MB132 | Mbeya | *Pseudocercospora fijiensis* | XM_007925807 | 0 | 95.6 | 84.6 |
| 22 | MB22 | Mbeya | *Pseudocercospora fijiensis* | XM_007925807 | 0 | 94.1 | 82.6 |
| 23 | MB12 | Mbeya | *Pseudocercospora fijiensis* | XM_007925807 | 0 | 92.5 | 92.8 |
| 24^a^ | MR9 | Morogoro | *Pseudocercospora fijiensis* | XM_007925807 | 3.04E-163 | 89.7 | 65.8 |
| 25^a^ | MR10 | Morogoro | *Pseudocercospora fijiensis* | XM_007925807 |  | 93.9 | 72.7 |
| 26^a^ | MR18 | Morogoro | *Pseudocercospora fijiensis* | XM_007925807 |  | 97.8 | 82.2 |

^a^ Known *Pseudocercospora fijiensis* isolates previously isolated from Morogoro were included as a reference point.
